# Commentary: Trends and Early Complications in Direct-to-Implant Breast Reconstruction: An Updated Analysis of the ACS-NSQIP Database

**DOI:** 10.1177/22925503231163243

**Published:** 2023-03-13

**Authors:** Mitchell H. Brown

**Affiliations:** 1 Department of Surgery, 7938 University of Toronto, Toronto, Canada

It is a pleasure to provide a brief commentary on the article “Trends and Early Complications in Direct-to-Implant Breast Reconstruction: An Updated Analysis of the ACS-NSQIP Database” by Plotsker et al.^1^ The authors are an accomplished group from a very busy cancer center with extensive experience in post-mastectomy breast reconstruction. The two primary goals of this article were to analyse early (30 day) complication rates following immediate direct-to-implant (DTI) breast reconstruction and to assess the change in DTI rates in mastectomy patients over a ten-year period from 2010 to 2019.

The authors chose to group early complications into two main categories: major surgical complications and medical complications. Data was obtained from the American College of Surgeons – National Surgical Quality Improvement Program (ACS-NSQIP) and was compared to a previously published study by Wink et al^2^ which analysed similar data between 2005 and 2010. The primary findings were a major surgical 30-day complication rate of 10%, a medical 30-day complication rate of 0.83%, and a trend in DTI reconstruction rates from 5.2% in 2010 to 15.1% in 2019. The authors point out that the early complication rate was similar to that reported by Wink from 2005 to 2010 (9%). Specific risk factors linked to complications were elevated BMI, history of smoking, hypertension, blood disorders, and intraoperative blood transfusion.

With alloplastic breast reconstruction being the predominant option for postmastectomy breast reconstruction, along with the increasing trend towards DTI procedures, this updated data from a comprehensive national database is important and welcome information to be used during patient selection as well as informed consent. Of note, the major surgical complication rate was largely stable between 9% and 10% comparing data over the course of 15 years. One is left to wonder why advances such as improved attention to mastectomy technique, intraoperative assessment of tissue perfusion, and increasing use of acellular dermal matrices, has not positively impacted this rate. Perhaps those advances have been offset by other changes such as an increased percentage of nipple-sparing mastectomies or greater usage of the prepectoral plane. Further research will be necessary to tease out the impact of these changing trends.

The authors provide a description of the limitations of this study, many of which are unavoidable when performing an analysis on an existing database. Several of these are worth further discussion. The type of mastectomy is recorded as radical, simple, or subcutaneous. Specific variables such as nipple sparing, skin sparing, skin reduction, and therapeutic or prophylactic mastectomy rates within this population are not included and this will impact the generalizability of the data within today's current practices. Similarly, the pocket location may be impactful on the rate and type of complication following implant placement. This is particularly relevant when comparing data over the past 15 years.

It is noteworthy that the rates of DTI reconstruction tripled during the study period. Although not captured by this study, it would be useful to understand how this finding compares to overall rates of reconstruction postmastectomy as well as rates of planned two stage alloplastic and autogenous reconstruction. Perhaps this will be a focus of future research. The authors should be congratulated on a valuable contribution to better understanding outcomes in alloplastic breast reconstruction.
